# Simultaneous Determination of Ferulic Acid and Phthalides of *Angelica Sinensis* Based on UPLC-Q-TOF/MS

**DOI:** 10.3390/molecules20034681

**Published:** 2015-03-13

**Authors:** Wen-Long Wei, Lin-Fang Huang

**Affiliations:** Institute of Medicinal Plant Development, Chinese Academy of Medical Sciences & Peking Union Medical College, Beijing 100193, China; E-Mail: 13521032532@163.com

**Keywords:** ultra-performance liquid chromatography quadrupole time-of-flight tandem mass spectrometry, *Angelica sinensis*, geoherbalism, fingerprint

## Abstract

The radix of *Angelica sinensis* (AS) is one of the most commonly used as a herbal medicine. To investigate the geoherbalism and quality evaluation of AS, an ultra performance liquid chromatography coupled with electrospray ionization quadrupole time-of-flight tandem mass spectrometry (UPLC-ESI-Q-TOF/MS) method was established to analyze and identify ferulic acid and phthalides in AS. The results showed that among samples collected in four regions, the relative contents of ferulic acid and phthalides were highest in samples collected in Gansu, and the samples from the four different regions were apparently classified into four groups. Meanwhile, the relative content in non-fumigated root was higher than after sulfur-fumigation and the sulfur-fumigated and non-fumigated samples were obviously divided into two groups by PCA. The paper establishes a systematic and objective evaluation system to provide a scientific basis for evaluating the quality of AS.

## 1. Introduction

*Angelica sinensis* (AS; Danggui in Chinese), one of the most important traditional Chinese medicinal herbs, is used for tonifying blood and treating female irregular menstruation and amenorrhoea [1,2,3]. Seventy formulae are recorded to contain AS in China, and 56 formulae in Japan contain AS [1,4]. Besides its common use in Asia, AS is also used as a health food product for women’s care in Europe and America [5]. AS, an important medicinal resource, is mainly produced in the Chinese provinces of Gansu, Yunnan, Sichuan, and Hubei. The major compounds of AS are organic acids, phthalides, and polysaccharides. Ferulic acid and ligustilides serve as chemical markers to assess the quality of AS plants [6]. Ferulic acid inhibits platelet aggregation, whereas ligustilide demonstrates anti-asthmatic activity [7,8]. Studies have also been published assessing the effects and possible mechanisms of action of AS polysaccharides against carbon tetrachloride-induced liver injury [9].

A number of studies on the pharmacology and phytochemistry of AS have been conducted. However, few studies have investigated its geoherbalism and quality. Geographical indications played an important role in regional development and quality evaluation of Chinese herbal medicines [10]. Qian *et al.* reported the fingerprinting of AS from different growth periods by UPLC-TOFMS and chemometrics [11]. Wedge *et al.* established the chemical fingerprinting of the bioactivity-guided fractionation of AS and *Angelica archangelica* root components by GC-MS [12]. In the present study, the UPLC-Q-TOF/MS method was applied to simultaneously identify ferulic acid and phthalides of samples from four different regions and processed samples, and profile samples of AS coupled with PCA. This is the first report on the simultaneous qualitative and quantitative analysis of ferulic acid and phthalides in AS using UPLC-Q-TOF/MS.

## 2. Results and Discussion

### 2.1. Optimization of Detection Wavelength

To detect chemical compounds in AS, several preliminary experiments were performed. For the optimization of detection wavelength, different detection wavelengths such as 261 nm, 281 nm and 321 nm were checked. UPLC with ultraviolet detection was used to analyze reference solutions of ferulic acid, senkyunolide A, *n*-butylphthalide, ligustilide, and butylidenephthalide.

**Figure 1:**
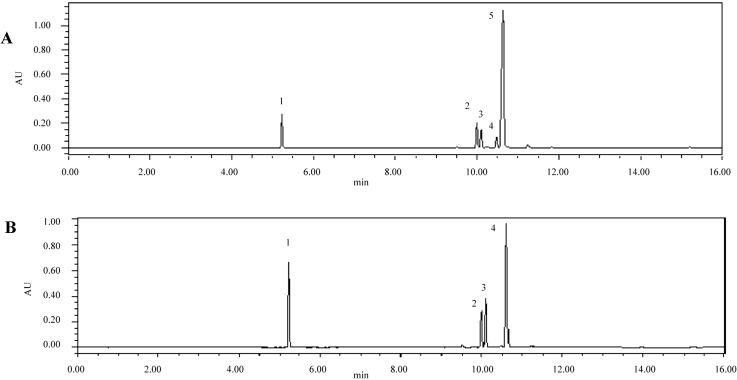
The UPLC-PDA chromatogram of ferulic acid and phthalides in *Angelica sinensis* by UV at 261 nm (**A**), 281 nm (**B**); Peaks: (1) Ferulic acid; (2) Senkyunolide A; (3) *n*-Butylphthalide; (4) Ligustilide; (5) Butylidenephthalide.

The maximum absorption wavelength of ferulic acid and ligustilide was 321 nm. The maximum absorption wavelength of senkyunolide A was 281 nm, and that of *n*-butylphthalide and butylidene- phthalide was 261 nm. Ferulic acid, senkyunolide A, *n*-butylphthalide, and ligustilide also exhibited a high response at 281 nm (Figure 1). By contrast, the response of butylidenephthalide at 281 nm was only 1/10 of that at 261 nm. The best separation was thus achieved with detection wavelengths of 261/281 nm, and a column temperature of 25 °C at a flow rate of 0.1 mL/min. These optimized conditions were further developed for resolution, baseline and analysis time.

### 2.2. UPLC Fingerprints

**Figure 2:**
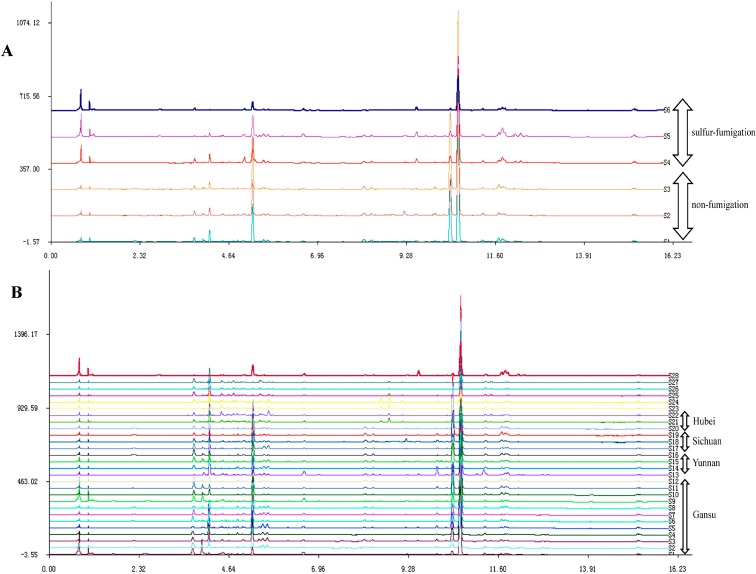
The fingerprint of non-fumigated and sulfur-fumigated *Angelica sinensis* (**A**) and *Angelica sinensis* from different regions (**B**) by UPLC. Peaks: (1) Ferulic acid; (2) Senkyunolide A; (3) n-Butylphathlide; (4) Ligustilide.

A total of 34 samples were analyzed using UPLC according to the experimental conditions for fingerprint analysis [12,13]. These samples corresponded to various sources and conditions, which included different growing areas and different processing methods. Using the software “Similarity Evaluation System for Chromatographic Fingerprint of TCM”, the median chromatogram and correlation coefficients of Gansu, Hubei, Yunnan, Sichuan samples and processed samples could be determined. The fingerprints of 28 samples from four different regions are shown in Figure 2B, and six processed samples with the standard samples are shown in Figure 2A.

As shown in Figure 2, peaks in 34 samples had reasonable height and good resolutions and were assigned as the “characteristic peaks” for the identification of AS. The absorption intensity of some peaks clearly differed. This observation indicated that these chromatograms were associated with similar chemical components of AS. The characteristic peaks of samples in the fingerprints were compared and the relative content of ferulic acid and phthalides of samples from different regions, namely Hubei, Sichuan, Yunnan and Gansu, were obviously different. The compounds in AS from different regions showed diversity, and the relative contents of ferulic acid and phthalides of samples collected in Gansu were the highest, indicating that the quality of AS from Gansu was the best among the four regions. In addition, the relative contents in non-fumigated and sulfur-fumigated samples were different, and the relative content of ferulic acid and phthalides in sulfur-fumigation samples was less than in non-fumigated ones, suggesting that the quality with no fumigation was better than with sulfur fumigation. In Figure 2, it can be seen that the prominent peaks that represent ferulic acid and the phthalides were the major peaks in AS, so these peaks which were chosen as the major characteristic peaks that could potentially be used as marker peaks for the quality control and geo-herbalism evaluation of AS.

### 2.3. Identification of the Major Peaks in AS by UPLC-Q-TOF/MS Analysis

**Figure 3:**
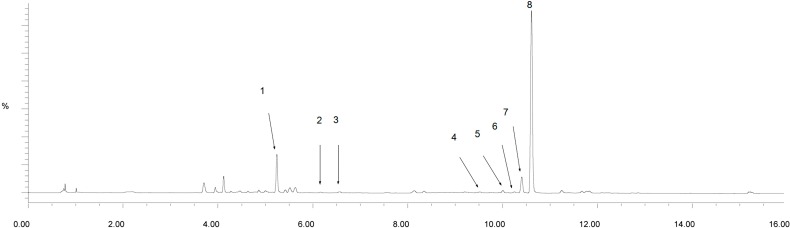
The total ion chromatogram of UPLC/Q-TOF-MS of *Angelica sinensis*. Peaks: (1) Ferulic acid; (2) Senkyunolide H/I; (3) Senkyunolide I /H; (4) Coniferyl ferulate; (5) Senkyunolide A; (6) *n*-Butylphthalide; (7) Ligustilide; (8) Butylidenephthalide.

Under the present chromatographic and MS conditions, a total of eight major peaks were detected in the two kinds of sample solutions. The identities of the major peaks were identified or tentatively assigned by comparison of their t_R_, UV_λmax_, and MS fragments with the reference standards and information from the literature or by matching the empirical molecular formulae with those of the published components of AS [14,15,16]. The total ion chromatogram is presented in Figure 3.

The retention time and precise molecular weight of peaks 1, 5, 6, 7, and 8 were consistent with the standard samples, and they were identified as ferulic acid, senkyunolide A, and *n*-butylphthalide. Similarly, the retention time and precise molecular weight of peaks 2, 3, and 4 were consistent with the literature data [16], and they were identified as senkyunolide H/I, senkyunolide I/H, and coniferyl ferulate. The analyzed and identified compounds are listed in Table 1.

**Table 1:** Analysis of *Angelica sinensis* by UPLC/Q-TOF-MS.

Peak no.	t_R_ (min)	Assigned Identity	Molecular Fomular	Mean Measured Mass (Da)	Theoretical Exact Mass (Da)	ppm
1	5.26	Ferulic acid	C_10_H_10_O_4_	195.0657	195.0659	1.0
2	6.17	Senkyunolide H/I	C_12_H_16_O_4_	225.1121	225.1127	−2.7
3	6.54	Senkyunolide I /H	C_12_H_16_O_4_	225.1124	225.1127	−1.3
4	9.51	Coniferyl ferulate	C_20_H_20_O_6_	357.1367	357.1338	8.1
5	10.01	Senkyunolide A	C_12_H_16_O_2_	193.1259	193.1229	15.5
6	10.30	*n*-Butylphthalide	C_12_H_14_O_2_	191.1064	191.1072	−4.2
7	10.46	Ligustilide	C_12_H_14_O_2_	191.1069	191.1072	−4.2
8	10.59	Butylidenephthalide	C_12_H_12_O_2_	189.0909	189.0916	−3.7

It was reported that ferulic acid could inhibit platelet aggregation, increase coronary blood flow, relax or stimulate smooth muscle and possesses anti-arrhythmic effect [17]. More recent study found that phthalides inhibited the formation of reactive oxygen species and lipid peroxidation, and enhanced the cellular resistance to hydrogen peroxide-induced oxidative damage [18]. Therefore, it might be more reasonable to choose ferulic acid and phthalides as characteristic marker compounds for the quality control of AS.

### 2.4. Quantification of the Five Main Compounds in AS

UPLC-Q-TOF/MS was applied for the simultaneous quantification of five chemical compounds, including ferulic acid and phthalides, in 34 samples. The content of these chemical compounds is shown in Table 2, and the data were analyzed by statistical methods.

**Figure 4:**
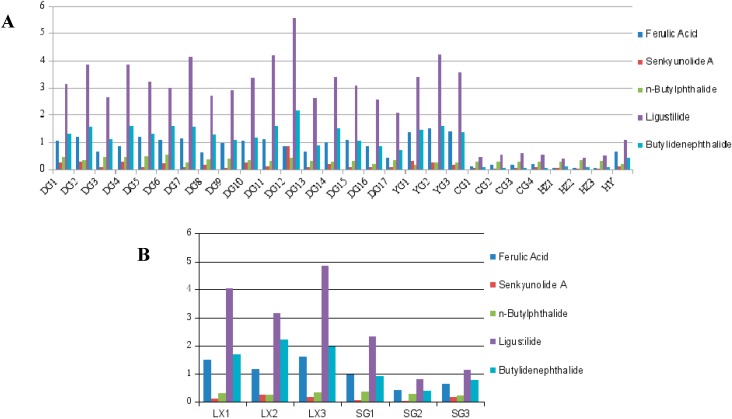
The content of ferulic acid and phthalides in *Angelica sinensis* from different regions (**A**) and processed *Angelica sinensis* (**B**). Non-fumigated: LX1, LX2, LX3; sulfur-fumigation: SG, SG2, SG3.

As shown in Table 2 and Figure 4A, among the 28 different samples the highest amounts of all the compounds (ferulic acid and phthalides) among the four regions were detected in Gansu samples, and the total contents in samples from Gansu and Yunnan were higher than those in the Sichuan and Hubei samples. All sample from the four locations showed different concentrations of ferulic acid and phthalides. The ferulic acid content in Yunnan samples was the highest, which was higher than the 0.05% provision of the 2010 version of the Chinese Pharmacopoeia. In Gansu samples, except DG17, the contents of the remaining samples were higher than that of the Pharmacopoeia standard. Moreover, the ferulic acid content was slightly lower in Gansu samples than that in Yunnan samples, which conformed to the Pharmacopoeia values. The ferulic acid contents in Sichuan and Hubei were far below the Pharmacopoeia levels. The samples in Sichuan were only one-fifth of those mentioned in the Pharmacopoeia. The samples in Hubei were only 1/50 of those in the Pharmacopoeia, so they were regarded as unqualified products. The total phthalide content of samples in Gansu was higher than that in Yunnan, and the phthalide contents of samples in Sichuan and Hubei were lower than that in Yunnan. The butylidenephthalide content of samples in Gansu was the highest among the four regions. The contents of senkyunolide A and *n*-butylphthalide were relatively lower than those of the other compounds, and the total contents of senkyunolide A and *n*-butylphthalide of samples from Gansu were the highest. The content of main biochemical compounds is an important criterion factor in the evaluation of the geoherbalism of Chinese herbal medicines, and the results indicate that the concentration differences of ferulic acid and phthalides could be used for evaluating the quality and geoherbalism of AS.

**Table 2:** Contents of ferulic acid and phthalides in *Angelica sinensis*.

Sample	Content (%)					Total Content (%) (mg/g)
	Ferulic Acid (mg/g)	Senkyunolide A (mg/g)	n-Butylphthalide (mg/g)	Ligustilide (mg/g)	Butylidenephthalide (mg/g)	
DG1	1.061	0.226	0.442	3.121	1.306	6.156
DG2	1.191	0.261	0.317	3.838	1.559	7.166
DG3	0.666	0.104	0.454	2.647	1.117	4.988
DG4	0.839	0.282	0.445	3.831	1.597	6.994
DG5	1.198	0.086	0.468	3.215	1.309	6.276
DG6	1.094	0.215	0.551	3.002	1.589	6.451
DG7	1.128	0.098	0.226	4.128	1.559	5.118
DG8	0.622	0.141	0.365	2.695	1.295	5.401
DG9	0.968	0.066	0.383	2.919	1.065	6.193
DG10	1.056	0.252	0.338	3.373	1.174	7.318
DG11	1.100	0.113	0.311	4.193	1.601	9.773
DG12	0.836	0.829	0.411	5.551	2.146	6.33
DG13	0.653	0.090	0.311	2.596	0.865	5.606
DG14	0.978	0.189	0.258	3.396	1.509	6.33
DG15	1.085	0.077	0.310	3.081	1.053	5.606
DG16	0.842	0.099	0.194	2.540	0.847	4.522
DG17	0.405	0.101	0.340	2.060	0.717	3.623
YG1	1.364	0.301	0.153	3.379	1.431	6.628
YG2	1.496	0.235	0.244	4.243	1.603	7.821
YG3	1.389	0.142	0.226	3.582	1.362	6.701
CG1	0.113	0.055	0.255	0.440	0.097	0.96
CG2	0.144	0.039	0.255	0.537	0.047	1.022
CG3	0.150	0.052	0.258	0.588	0.065	1.113
CG4	0.185	0.092	0.270	0.552	0.051	1.15
HZ1	0.059	0.054	0.284	0.400	0.121	0.918
HZ2	0.056	0.020	0.343	0.431	0.103	0.953
HZ3	0.049	0.019	0.313	0.497	0.083	0.961
HY	0.646	0.109	0.180	1.076	0.420	2.422
LX1	1.502	0.115	0.335	4.041	1.701	7.694
LX2	1.186	0.26	0.255	3.155	2.202	7.058
LX3	1.619	0.16	0.357	4.847	1.974	8.957
SG1	0.973	0.058	0.377	2.324	0.94	4.672
SG2	0.433	0.025	0.299	0.821	0.411	1.989
SG3	0.656	0.171	0.225	1.137	0.785	2.974

The contents of ferulic acid and phthalides in sulfur-fumigated and non-fumigated AS are shown in Figure 4B. From Table 2 and Figure 4B, and with non-fumigated samples used as a reference, the content of five major constituents (ferulic acid and phthalides) in sulfur-fumigated AS were found to be significantly reduced. This result is probably due to changes in the components resulting from the sulfur-fumigation process, which in turn affects the medicinal properties. The concentrations of ferulic acid and phthalides was one of most important parameters associated with the quality of processed AS, so sulfur fumigation should be controlled strictly to guarantee the quality of medicines and also due to fact the residual sulphur dioxide from sulfur fumigation is harmful to the health.

### 2.5. Principal Component Analysis (PCA)

In order to discriminate between AS from the top geoherbal region and that from non-top geoherbal regions, multivariate statistical software such as SPSS (SPSS for Windows 9.0, SPSS Inc., Chicago, IL, USA) and SIMCA-P (Umetrics, Umea, Sweden) were employed. PCA is an unsupervised clustering method requiring little prior knowledge of the data set and acts to reduce the dimensionality of multivariate data without losing important information [19].

**Figure 5:**
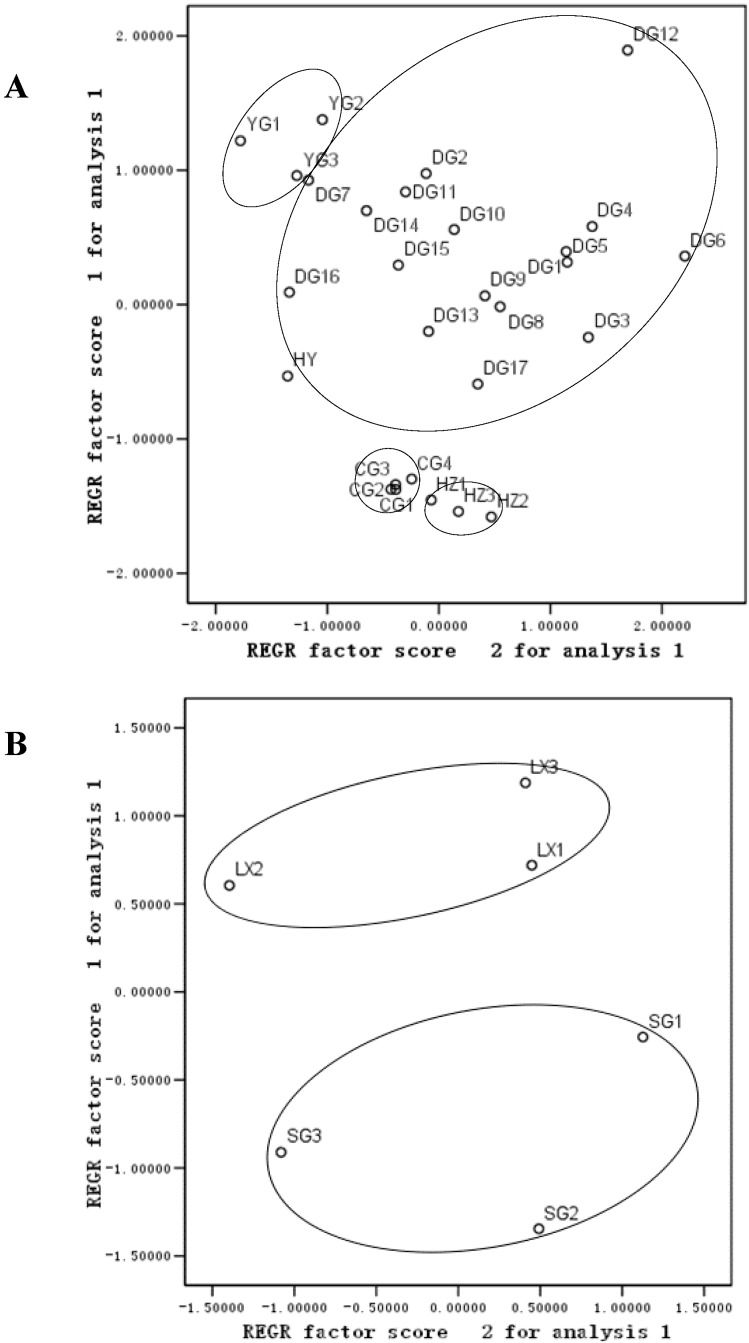
Analysis of the chemical content of *Angelica sinensis* from different regions (**A**) and processed *Angelica sinensis* (**B**) based on PCA.

PCA classification using the original data obtained from content analysis was applied to evaluate the differences among AS samples. With five principle components, explaining the variation in data, some origins were grouped separately. PCA score plots were plotted using mean-centered par-scaled data. The statistical results of SPSS are shown in Figure 5A. Figure 5B shows the PCA score plot of 28 AS samples from different origins. A clear separation of the four different groups and two groups was observed in the PCA scores plot, where each coordinate represents different origins. Most of the AS samples from Gansu and Yunnan were clustered along the negative side of PC1, while those from Sichuan and Huibei were gathered in the positive region. This suggested that there were differences in the metabolite profiling of AS samples due to variation in the product quality according to the sample origin. The AS samples from a wide variety of origins (Gansu, Yunnan, Hubei, and Sichuan) were cluttered in four groups. The scattered dots representing different origins were disparate, which indicated that within each group, the quality of AS samples from each of these origins was relatively unstable and exhibited great variations in terms of the content of ferulic acid and phthalides. PCA analysis confirmed that the contents of ferulic acid and phthalides in AS were different among the four regions, thereby explaining the diversity of quality among the different geographic patterns. The dispersed distribution of some of the AS samples from different regions might be caused by the differences in specific growth environments, such as climatic conditions, elevation, and soil environment, which should be further investigated.

The processed samples were analyzed using optimized chemical fingerprinting and PCA analysis. Statistical analysis was conducted using SPSS16.0, and the results are shown in Figure 5B. Sulfur-fumigated and non-fumigated AS were clearly distinguished by PCA analysis as the contents of ferulic acid and phthalides showed diversity between sulfur-fumigated and non-fumigated samples. Considering that the safety of sulfur-fumigated AS has not been systematically evaluated, the government should strengthen supervision to prevent the consumption of sulfur-fumigated AS from to ensure the efficacy, safety, and quality consistency of AS. A safe and efficient alternative method for the store and preservation of AS should also be developed.

## 3. Experimental Section

### 3.1. Chemicals, Reagents and Materials

A Waters Acquity UPLC PDA (Waters Co., Milford, MA, USA), including four high-pressure gradient pumps, a vacuum degassing machine, automatic sampler, and and Empower 2 chromatographic work station, an electronic analytical balance (AB135-S, Mettler, Mettler Toledo Inc., Columbus, OH, USA), electrically-heated thermostatic water bath (Beijing Analysis Instrument Factory, Beijing, China), ELGA PURELAB Classic-UVF water meter (Company, High Wycombe, UK), and SPSS16.0 software were used in this study.

**Figure 6:**
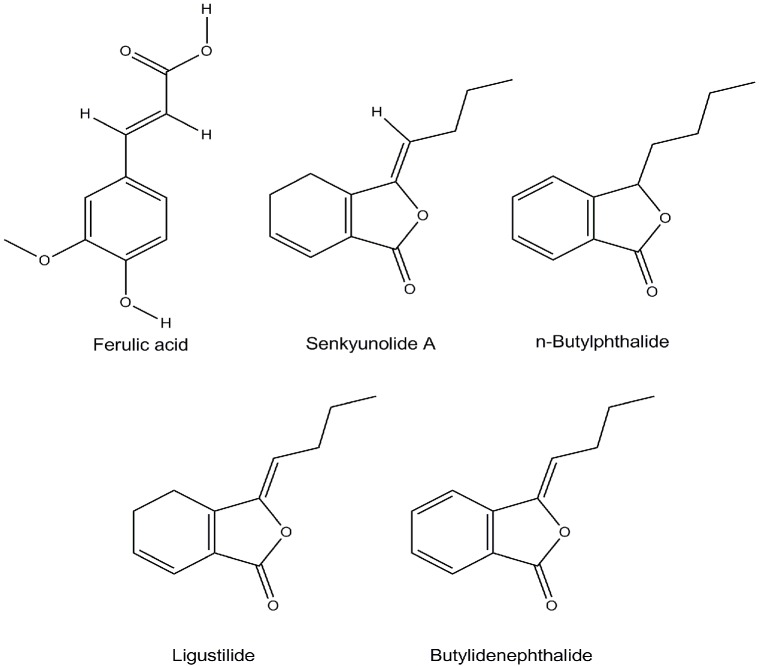
Chemical structures of ferulic acid and phthalides.

Acetonitrile (chromatographically pure, Fisher, Beijing, China), methanoic acid (chromatographically pure, Fisher), and methanol were analytically pure (Beijing Chemical Plant, Beijing, China). Pure water was synthesized in the laboratory. The reference material of ferulic acid (lot number: 110773-201012) was purchased from the National Institute for the Control of Pharmaceutical and Biological Products (Beijing, China). Ligustilide (lot number: MUST-11072416), *n*-butylphthalide (lot number: MUST-12020706), butylidenephthalide (lot number: MUST-12071103), and senkyunolide A (lot number: MUST-10102309) were purchased from Chengdu International Biotechnology Co. Ltd. (Sichuan, China). Their purities were above 98% as determined by HPLC analysis. The chemical structures is shown in Figure 6. Experimental materials are described in Table 3.

#### 3.1.1. UPLC Conditions

Analysis was performed using an ACQUITY UPLC system with a conditioned autosampler at 4 °C. Separation was carried out on a BEH C 18 column (100 mm × 2.1 mm i.d., 17 µm) [20]. The column temperature was maintained at 35 °C. The column was eluted with a gradient mobile phase of acetonitrile (solvent system A) and 0.1% methanoic acid in ultra-pure water (solvent system B). Gradient elution was performed as follows: 0–2 min: 100%–95% B; 2–4 min: 95% B; 4–7 min: 95%–76% B; 7–8 min: 76%–72% B; 8–10 min: 72%–50% B; 10–12 min: 50%–30% B; 12–14 min: 30%–0% B; 14–15 min: 0% B; and 15–16 min: 0%–95% B. Methanol-water with a flow rate of 1 mL/min provided a much better resolution than acetonitrile-water, and the injection volume was 10 μL.

**Table 3:** *Angelica sinensis* used in the experiments.

	Sample Source	Collection Time	Description
DG1	Zhifang Village, Sigou Xiang, Min Xian, Gansu	21 October 2012	Biennial
DG2	Zhadi Village, Sigou Xiang, Min Xian, Gansu	21 October 2012	Biennial
DG3	Babu Village, Sigou Xiang, Min Xian, Gansu	22 October 2012	Biennial
DG4	Lvye Village, Mazichuan Xiang, Min Xian, Gansu	22 October 2012	Biennial
DG5	Pashi Village, Awu Xiang, Dangchang Xian, Gansu	22 October 2012	Biennial
DG6	Wendou Village, Meichuan Country, Min Xian, Gansu	23 October 2012	Biennial
DG7	Wendou Village, Meichuan Country, Min Xian, Gansu	24 October 2012	Biennial
DG8	Lizhu Village, Sigou Xiang, Min Xian, Gansu	24 October 2012	Biennial
DG9	Qijiazhuang She, Luming Village, Wuzhu Xiang, Weiyuan Xian, Gansu	24 October 2012	Biennial
DG10	Shengbutan She, Hadiwo Village, Huichuan, Weiyuan Xian, Gansu	25 October 2012	Biennial
DG11	Huichuan Countr, Weiyuan Xian, Gansu	25 October 2012	Biennial
DG12	Shitou Zhuang, Wuzhu Xiang, Weiyuan Xian, Gansu	25 October 2012	Biennial
DG13	Wuzhu Village, Wuzhu Xiang, Weiyuan Xian, Gansu	25 October 2012	Biennial
DG14	Shitou Zhuang, Wuzhu Xiang, Weiyuan Xian, Gansu	25 October 2012	Biennial
DG15	Wuzhu Village, Wuzhu Xiang, Weiyuan Xian, Gansu	25 October 2012	Biennial
DG16	Huichuan Zhen, Weiyuan Xian, Gansu	26 October 2012	Biennial
DG17	Xiadiema Village, Qingshui Xiang, Min Xian, Gansu	27 October 2012	Biennial
YG1	Machang Village, Caohai Country, Heqing Xian, Yunnan	10 November 2012	Biennial
YG2	Machang Village, Caohai Country, Heqing Xian, Yunnan	10 November 2012	Biennial
YG3	Machang Village, Caohai Country, Heqing Xian, Yunnan	10 November 2012	Biennial
CG1	Shimeishan Village, Shiquan Xiang, Zhongjiang Xian, Deyang City, Sichuan	17 November 2012	Biennial
CG2	Shimeishan Village, Shiquan Xiang, Zhongjiang Xian, Deyang City, Sichuan	17 November 2012	Biennial
CG3	Shimeishan Village, Shiquan Xiang, Zhongjiang Xian, Deyang City, Sichuan	17 November 2012	Biennial
CG4	Shimeishan Village, Shiquan Xiang, Zhongjiang Xian, Deyang City, Sichuan	17 November 2012	Biennial
HZ1	Hongta Xiang, Chengguan Country, Fang Xian, Shiyan City, Hubei	20 November 2012	Biennial
HZ2	Hongta Xiang, Chengguan Country, Fang Xian, Shiyan City, Hubei	20 November 2012	Biennial
HZ3	Hongta Xiang, Chengguan Country, Fang Xian, Shiyan City, Hubei	20 November 2012	Biennial
HY	Hongta Xiang, Chengguan Country, Fang Xian, Shiyan City, Hubei	20 November 2012	Wild
LX1	Medicine market, Min Xian, Gansu	27 October 2012	sulfur-fumigation
LX2	Medicine market, Min Xian, Gansu	27 October 2012	sulfur-fumigation
LX3	Medicine market, Min Xian, Gansu	27 October 2012	sulfur-fumigation
SG1	Medicine market, Min Xian, Gansu	27 October 2012	air-dried
SG2	Medicine market, Min Xian, Gansu	27 October 2012	air-dried
SG3	Medicine market, Min Xian, Gansu	27 October 2012	air-dried

#### 3.1.2. MS Conditions

MS/MS was performed on a Q-TOF mass spectrometer equipped with an ESI source (Micromass, Manchester, UK). The nebulization gas flow rate was set at 500 L/h at 500 °C, the cone gas was set to 30 L/h, and the source temperature was set at 100 °C [21]. The capillary and cone voltages were set at 3000 and 40 V, respectively. The Q-TOF acquisition rate was 0.2 s. A lock mass of leucine enkephalin for positive ESI mode (*m/z* = 556.2771) and negative ESI mode (*m/z* = 554.2615) was used via a LockSpray^TM^ interface. Mass accuracy and reproducibility were maintained. The MS data were collected in centroid mode from *m/z* 50 to 1200 to ensure accurate mass measurements over a wide dynamic range. Dynamic range enhancement was applied throughout the MS experiment. For the precursor ions and fragment, the accurate mass and composition ions were calculated using MassLynx V4.1 software (Waters Co.) included with the instrument. The ESI interface was operated in the positive and negative ion modes.

#### 3.1.3. Preparation of the Sample Solutions

The crude drugs were milled into a powder and oven dried at 25 °C until the weight remained constant. Each of the finely powdered samples (0.2 g) was accurately weighed and dissolved in 20 mL of 70% methanol. The mixture was precisely weighed and heated to reflux for 30 min, cooled, and weighed again. Subsequently, 70% methanol was added to compensate for the lost weight during the reflux and then mixed well. The sample solution was filtered through a 0.22 μm membrane filter before it was injected into the UPLC system for analysis.

#### 3.1.4. Preparation of the Standard Solutions

The standard solutions of ferulic acid, senkyunolide A, *n*-butylphthalide, ligustilide, and butylidenephthalide were prepared by dissolving accurately weighed powders in 70% methanol to obtain a final concentration of 0.5 mg/mL. The solutions were stored in a refrigerator at 4 °C before analysis.

### 3.2. Method Validation

The method was validated for its linearity, limit of detection (LOD), limit of quantification (LOQ), precision, repeatability, and recovery [22]. Linearity was examined using the standard solutions. A mixed stock solution consisting of the five analyses was prepared and diluted to the appropriate concentrations for the construction of calibration curves. Each calibration curve was injected in triplicate. The calibration curves were constructed by plotting the peak area (Y) *versus* the concentrations (X, mg/mL) of the mixed standard solutions, which were expressed by the equation given in Table 2. All calibration curves showed good linear regressions (R2 ≥ 0.9991) within the test ranges. LODs and LOQs are frequently calculated by the signal-to-noise ratio (S/N) in chromatographic techniques. Generally, LOD is calculated at the S/N ratio of 3, and LOQ is determined at the S/N of 10. The sensitivity of instrumental methods can be easily expressed by instrumental LOD/LOQ estimated using this approach. However, factors influencing these limits (such as matrix effects and recoveries) might become overestimated. Intra- and inter-day variations were utilized to determine the precision of the developed method.

The intra-and inter-day variations of the method were determined by analyzing the six replicate samples (sample DG1) within 1 d and on three consecutive days, respectively. The RSD values of the retention time and peak area of the five compounds in the inter-day variations were 0.24%–0.72% and 1.07%–3.34%, respectively, and the RSD values of the retention time and peak area of the five compounds in the intra-day variations were 0.11%–0.44% and 0.86%–3.51%, respectively. These findings showed that the precision of the instrument was good. To confirm the repeatability of the method, six independently prepared solutions from the same sample (DG1) were analyzed. The RSD values of the peak area were between 0.61% and 2.89%. The stabilities of the sample solutions were analyzed after storage for 0, 2, 4, 8, 12, 24, and 36 h at room temperature. The sample solution was stable within 36 h (RSD: 0.76%–2.25%). A recovery test was used to evaluate the accuracy of the method. The standards of five compounds were spiked into the samples (0.2 g, DG1), extracted, processed, and quantified in accordance with the established procedures. The average recoveries of the five compounds were 98.33%–102.22%, with RSD values between 1.16% and 4.72%. These validation results indicated that the conditions used in the quantitative determination of the target compounds were acceptable.

## 4. Conclusions

In the present study, the contents of ferulic acid and phthalides were determined simultaneously by UPLC-Q-TOF/MS, and a fingerprint was established and content data was analyzed to determine geographical indications. Eight chemical compounds including ferulic acid, senkyunolide H, senkyunolide I, coniferyl ferulate, senkyunolide A, *n*-butylphthalide, ligustilide and butylidenephthalide in AS were identified within a relatively short time. The samples from different regions showed diversity, so attention should be paid to the origin of samples. Meanwhile, comparing the differences between sulfur-fumigated and non-fumigated AS, it is clear that sulfur-fumigation influences in a negative way the quality of this Chinese herbal medicine. The results indicated that AS from different regions could be discriminated rapidly, and ferulic acid and the phthalide compounds determined in the current study can be used for ensuring a scientific evaluation of the quality and geoherbalism of AS.
